# Supplementary material for: Single Cell Transcriptomic and Chromatin Profiles Suggest Layer Vb Is the Only Layer With Shared Excitatory Cell Types in the Medial and Lateral Entorhinal Cortex
Source: Front Neural Circuits. 2022 Jan 26;15:806154. doi: 10.3389/fncir.2021.806154 (PMC8826650; doi:10.3389/fncir.2021.806154)
Supplement: Supplementary file 14 [file Data_Sheet_1.ZIP › 20/knownResults.html]

20 - Homer Known Motif Enrichment Results


# Homer Known Motif Enrichment Results (20)

Homer *de novo* Motif Results  
Gene Ontology Enrichment Results  
Known Motif Enrichment Results (txt file)  
Total Target Sequences = 9663, Total Background Sequences = 38945

|  |  |  |  |  |  |  |  |  |  |  |  |
| --- | --- | --- | --- | --- | --- | --- | --- | --- | --- | --- | --- |
| Rank | Motif | Name | P-value | log P-pvalue | q-value (Benjamini) | # Target Sequences with Motif | % of Targets Sequences with Motif | # Background Sequences with Motif | % of Background Sequences with Motif | Motif File | SVG |
| 1 | C A T G A G T C G A C T C G T A C G A T G C A T G A C T G C A T C G A T C T A G C A T G T G A C | Mef2b(MADS)/HEK293-Mef2b.V5-ChIP-Seq(GSE67450)/Homer | 1e-409 | -9.421e+02 | 0.0000 | 2751.0 | 28.47% | 4692.9 | 12.05% | motif file (matrix) | svg |
| 2 | C G A T T G C A T G C A G A T C C G T A A C T G T G A C G A C T C A T G A C T G | Tcf21(bHLH)/ArterySmoothMuscle-Tcf21-ChIP-Seq(GSE61369)/Homer | 1e-340 | -7.844e+02 | 0.0000 | 2835.0 | 29.34% | 5379.2 | 13.81% | motif file (matrix) | svg |
| 3 | C A T G G T A C G A C T G C T A C G T A C G T A C G T A G C T A G A C T C T G A T C A G G T A C | Mef2c(MADS)/GM12878-Mef2c-ChIP-Seq(GSE32465)/Homer | 1e-334 | -7.692e+02 | 0.0000 | 1732.0 | 17.92% | 2445.9 | 6.28% | motif file (matrix) | svg |
| 4 | T C G A A G T C C G T A A T C G A T G C C G A T A C T G A G T C A G C T A C T G | Tcf12(bHLH)/GM12878-Tcf12-ChIP-Seq(GSE32465)/Homer | 1e-303 | -6.990e+02 | 0.0000 | 2724.0 | 28.19% | 5322.4 | 13.66% | motif file (matrix) | svg |
| 5 | G T A C G A C T C G T A C T G A T C G A C G T A G C T A C A G T C T G A T A C G | Mef2a(MADS)/HL1-Mef2a.biotin-ChIP-Seq(GSE21529)/Homer | 1e-303 | -6.978e+02 | 0.0000 | 1562.0 | 16.16% | 2183.2 | 5.60% | motif file (matrix) | svg |
| 6 | G T C A T G C A G C T A A G T C C G T A A C T G T G A C G C A T T C A G C A G T | Ap4(bHLH)/AML-Tfap4-ChIP-Seq(GSE45738)/Homer | 1e-299 | -6.899e+02 | 0.0000 | 3364.0 | 34.81% | 7337.3 | 18.83% | motif file (matrix) | svg |
| 7 | C A T G A G T C A G C T C G T A C G A T C G A T G C A T G C A T C G A T C T G A C A T G T G A C | Mef2d(MADS)/Retina-Mef2d-ChIP-Seq(GSE61391)/Homer | 1e-279 | -6.429e+02 | 0.0000 | 973.0 | 10.07% | 993.6 | 2.55% | motif file (matrix) | svg |
| 8 | C T G A T C A G A G T C C G T A A T C G A T G C C G A T A C T G A G T C G A C T A T C G A G T C | MyoD(bHLH)/Myotube-MyoD-ChIP-Seq(GSE21614)/Homer | 1e-260 | -6.008e+02 | 0.0000 | 2293.0 | 23.73% | 4376.8 | 11.23% | motif file (matrix) | svg |
| 9 | A C G T T C G A T C G A A G T C G T C A T A C G A T G C A C G T A C T G A G C T | Myf5(bHLH)/GM-Myf5-ChIP-Seq(GSE24852)/Homer | 1e-243 | -5.606e+02 | 0.0000 | 2141.0 | 22.16% | 4068.0 | 10.44% | motif file (matrix) | svg |
| 10 | C T A G T C G A T G A C A G T C C G T A A C T G G T A C A C G T A C T G A C T G | BHLHA15(bHLH)/NIH3T3-BHLHB8.HA-ChIP-Seq(GSE119782)/Homer | 1e-237 | -5.473e+02 | 0.0000 | 3502.0 | 36.24% | 8394.0 | 21.55% | motif file (matrix) | svg |
| 11 | T G A C A G T C C G T A A C T G G T A C A C G T A C T G A C G T G A C T G A T C | Twist2(bHLH)/Myoblast-Twist2.Ty1-ChIP-Seq(GSE127998)/Homer | 1e-230 | -5.319e+02 | 0.0000 | 4197.0 | 43.43% | 10888.3 | 27.95% | motif file (matrix) | svg |
| 12 | T C G A T C G A A G T C C G T A C T A G T A G C A C G T A C T G | MyoG(bHLH)/C2C12-MyoG-ChIP-Seq(GSE36024)/Homer | 1e-225 | -5.204e+02 | 0.0000 | 2737.0 | 28.32% | 6013.1 | 15.43% | motif file (matrix) | svg |
| 13 | T C A G A C G T T C G A T A G C A G T C C G T A A C T G G T A C A C G T A C T G A T C G A G T C | Atoh1(bHLH)/Cerebellum-Atoh1-ChIP-Seq(GSE22111)/Homer | 1e-196 | -4.534e+02 | 0.0000 | 2764.0 | 28.60% | 6387.4 | 16.40% | motif file (matrix) | svg |
| 14 | T C G A A C G T C A T G G C T A T A G C C G A T G T A C G C T A A C G T A T G C | AP-1(bZIP)/ThioMac-PU.1-ChIP-Seq(GSE21512)/Homer | 1e-196 | -4.514e+02 | 0.0000 | 1877.0 | 19.42% | 3669.7 | 9.42% | motif file (matrix) | svg |
| 15 | C T A G T C G A A C G T A C T G C G T A A T G C A C G T G T A C C G T A A G C T G A T C G T A C | Atf3(bZIP)/GBM-ATF3-ChIP-Seq(GSE33912)/Homer | 1e-189 | -4.366e+02 | 0.0000 | 1716.0 | 17.76% | 3258.6 | 8.36% | motif file (matrix) | svg |
| 16 | A C T G C A T G T C G A A C G T A C T G C G T A A T C G A C G T G T A C C G T A G A C T A G T C | Fos(bZIP)/TSC-Fos-ChIP-Seq(GSE110950)/Homer | 1e-189 | -4.363e+02 | 0.0000 | 1574.0 | 16.29% | 2862.2 | 7.35% | motif file (matrix) | svg |
| 17 | C A T G C T A G T C G A A C G T A C T G C G T A T A G C C G A T T G A C C G T A A G C T G A T C | Fra2(bZIP)/Striatum-Fra2-ChIP-Seq(GSE43429)/Homer | 1e-189 | -4.358e+02 | 0.0000 | 1359.0 | 14.06% | 2285.0 | 5.87% | motif file (matrix) | svg |
| 18 | C T A G T C G A G C A T C A T G G C T A T A G C C G A T G T A C C T G A A G C T | JunB(bZIP)/DendriticCells-Junb-ChIP-Seq(GSE36099)/Homer | 1e-185 | -4.280e+02 | 0.0000 | 1491.0 | 15.43% | 2660.8 | 6.83% | motif file (matrix) | svg |
| 19 | A C T G C T A G T C G A C G A T C A T G G C T A A T C G C G A T G T A C G C T A A G C T G T A C | Fra1(bZIP)/BT549-Fra1-ChIP-Seq(GSE46166)/Homer | 1e-177 | -4.098e+02 | 0.0000 | 1487.0 | 15.39% | 2704.1 | 6.94% | motif file (matrix) | svg |
| 20 | C T A G T C G A A C G T A C T G C G T A T A G C C G A T G T A C C G T A A G C T G A T C G T A C | Jun-AP1(bZIP)/K562-cJun-ChIP-Seq(GSE31477)/Homer | 1e-177 | -4.093e+02 | 0.0000 | 828.0 | 8.57% | 1054.4 | 2.71% | motif file (matrix) | svg |
| 21 | C T A G T C G A C G A T A C T G C G T A T A C G A G C T T G A C G C T A A C G T G A T C T A G C | Fosl2(bZIP)/3T3L1-Fosl2-ChIP-Seq(GSE56872)/Homer | 1e-177 | -4.087e+02 | 0.0000 | 1030.0 | 10.66% | 1523.0 | 3.91% | motif file (matrix) | svg |
| 22 | C T A G A G T C T A C G T A C G T G A C C G T A A C T G T A G C G C A T C A T G A T G C A G C T | Ascl1(bHLH)/NeuralTubes-Ascl1-ChIP-Seq(GSE55840)/Homer | 1e-177 | -4.080e+02 | 0.0000 | 3410.0 | 35.29% | 8782.6 | 22.54% | motif file (matrix) | svg |
| 23 | T A C G T G C A A G T C C G T A A C G T T G A C A C G T A C T G A C T G G C A T | TCF4(bHLH)/SHSY5Y-TCF4-ChIP-Seq(GSE96915)/Homer | 1e-168 | -3.873e+02 | 0.0000 | 3544.0 | 36.68% | 9368.5 | 24.05% | motif file (matrix) | svg |
| 24 | C T G A T C A G G T A C G C T A A C T G T G A C G C A T C A T G | SCL(bHLH)/HPC7-Scl-ChIP-Seq(GSE13511)/Homer | 1e-167 | -3.851e+02 | 0.0000 | 7395.0 | 76.53% | 24720.7 | 63.45% | motif file (matrix) | svg |
| 25 | C A G T T G C A A C G T A C T G C G T A A T C G C G A T T G A C C G T A A C G T | BATF(bZIP)/Th17-BATF-ChIP-Seq(GSE39756)/Homer | 1e-166 | -3.842e+02 | 0.0000 | 1674.0 | 17.32% | 3317.8 | 8.52% | motif file (matrix) | svg |
| 26 | T A C G T A C G C T A G T C A G A G T C C G T A A T C G A T G C A C G T A C T G A G T C G A C T | Ascl2(bHLH)/ESC-Ascl2-ChIP-Seq(GSE97712)/Homer | 1e-156 | -3.598e+02 | 0.0000 | 2557.0 | 26.46% | 6159.6 | 15.81% | motif file (matrix) | svg |
| 27 | T G C A C T G A A T G C G T C A A C G T A T G C A C G T A C T G A C T G T G C A | ZBTB18(Zf)/HEK293-ZBTB18.GFP-ChIP-Seq(GSE58341)/Homer | 1e-145 | -3.346e+02 | 0.0000 | 1469.0 | 15.20% | 2903.6 | 7.45% | motif file (matrix) | svg |
| 28 | T C G A T G A C G T A C C G T A C A G T T G A C A C G T A C T G A G C T A G C T | NeuroG2(bHLH)/Fibroblast-NeuroG2-ChIP-Seq(GSE75910)/Homer | 1e-137 | -3.176e+02 | 0.0000 | 3438.0 | 35.58% | 9420.2 | 24.18% | motif file (matrix) | svg |
| 29 | C G T A T G A C T C G A A G T C C G T A A T C G A T G C A C G T A C T G A G T C | E2A(bHLH)/proBcell-E2A-ChIP-Seq(GSE21978)/Homer | 1e-135 | -3.121e+02 | 0.0000 | 2998.0 | 31.03% | 7910.8 | 20.31% | motif file (matrix) | svg |
| 30 | T C G A A G T C C G T A A T C G T A G C A C G T A C T G A G C T A C G T A G T C | Ptf1a(bHLH)/Panc1-Ptf1a-ChIP-Seq(GSE47459)/Homer | 1e-127 | -2.939e+02 | 0.0000 | 4911.0 | 50.82% | 15086.1 | 38.72% | motif file (matrix) | svg |
| 31 | A T G C T A C G A G C T T G C A C G T A C G A T A C G T C T G A | DLX5(Homeobox)/BasalGanglia-Dlx5-ChIP-seq(GSE124936)/Homer | 1e-127 | -2.936e+02 | 0.0000 | 1991.0 | 20.60% | 4661.9 | 11.97% | motif file (matrix) | svg |
| 32 | A T C G A T G C A G T C T A G C G A C T T C G A G C T A G C A T A G C T C T G A | DLX1(Homeobox)/BasalGanglia-Dlx1-ChIP-seq(GSE124936)/Homer | 1e-120 | -2.784e+02 | 0.0000 | 3087.0 | 31.95% | 8434.2 | 21.65% | motif file (matrix) | svg |
| 33 | A T C G A T C G A T G C G A C T T C G A C G T A G C A T A G C T C T G A T A C G | DLX2(Homeobox)/BasalGanglia-Dlx2-ChIP-seq(GSE124936)/Homer | 1e-119 | -2.754e+02 | 0.0000 | 3349.0 | 34.66% | 9385.8 | 24.09% | motif file (matrix) | svg |
| 34 | T G A C A T G C C G T A A T C G A T G C C A G T C A T G A C T G A G T C G T A C | HEB(bHLH)/mES-Heb-ChIP-Seq(GSE53233)/Homer | 1e-119 | -2.751e+02 | 0.0000 | 3636.0 | 37.63% | 10425.7 | 26.76% | motif file (matrix) | svg |
| 35 | T C A G C T A G A T G C G A C T T G C A C G T A A G C T A C G T C T G A T A C G | En1(Homeobox)/SUM149-EN1-ChIP-Seq(GSE120957)/Homer | 1e-116 | -2.690e+02 | 0.0000 | 3826.0 | 39.59% | 11167.6 | 28.67% | motif file (matrix) | svg |
| 36 | T C G A C A G T A C T G A G C T C G T A C G T A A C G T A C G T C T G A T A G C | Dlx3(Homeobox)/Kerainocytes-Dlx3-ChIP-Seq(GSE89884)/Homer | 1e-110 | -2.553e+02 | 0.0000 | 1704.0 | 17.63% | 3942.8 | 10.12% | motif file (matrix) | svg |
| 37 | C G A T T A C G T G A C G A C T C A T G C G T A T A C G A C G T G T A C C T G A | Bach2(bZIP)/OCILy7-Bach2-ChIP-Seq(GSE44420)/Homer | 1e-108 | -2.495e+02 | 0.0000 | 633.0 | 6.55% | 929.7 | 2.39% | motif file (matrix) | svg |
| 38 | A T G C T A C G A G T C G A C T T G C A C T G A G A C T A C G T C T G A T C A G | LHX9(Homeobox)/Hct116-LHX9.V5-ChIP-Seq(GSE116822)/Homer | 1e-80 | -1.849e+02 | 0.0000 | 2939.0 | 30.41% | 8596.7 | 22.07% | motif file (matrix) | svg |
| 39 | A G C T G T C A C G T A A C G T A C G T C T G A T C A G A T G C | Lhx2(Homeobox)/HFSC-Lhx2-ChIP-Seq(GSE48068)/Homer | 1e-78 | -1.818e+02 | 0.0000 | 2352.0 | 24.34% | 6540.9 | 16.79% | motif file (matrix) | svg |
| 40 | C G T A C G T A A G C T G A C T T G C A G T C A A C G T A G C T C T G A T C A G | Lhx3(Homeobox)/Neuron-Lhx3-ChIP-Seq(GSE31456)/Homer | 1e-76 | -1.752e+02 | 0.0000 | 3410.0 | 35.29% | 10404.1 | 26.71% | motif file (matrix) | svg |
| 41 | G A C T C T A G G A T C C A G T A C T G C T G A A T G C G C A T A T G C C T G A | MafA(bZIP)/Islet-MafA-ChIP-Seq(GSE30298)/Homer | 1e-73 | -1.700e+02 | 0.0000 | 1893.0 | 19.59% | 5050.4 | 12.96% | motif file (matrix) | svg |
| 42 | T C A G T G A C G T A C C G T A A C G T T G A C A C G T T C A G A G C T G A C T | NeuroD1(bHLH)/Islet-NeuroD1-ChIP-Seq(GSE30298)/Homer | 1e-71 | -1.658e+02 | 0.0000 | 1782.0 | 18.44% | 4705.6 | 12.08% | motif file (matrix) | svg |
| 43 | A C T G C A G T A C G T C G T A C G T A A C G T A C T G C T G A | Nkx6.1(Homeobox)/Islet-Nkx6.1-ChIP-Seq(GSE40975)/Homer | 1e-70 | -1.617e+02 | 0.0000 | 4749.0 | 49.15% | 15659.7 | 40.20% | motif file (matrix) | svg |
| 44 | C T G A T G A C T G A C C G T A A C G T T G A C A G C T C T A G A C G T G A C T | Olig2(bHLH)/Neuron-Olig2-ChIP-Seq(GSE30882)/Homer | 1e-67 | -1.546e+02 | 0.0000 | 3782.0 | 39.14% | 11999.8 | 30.80% | motif file (matrix) | svg |
| 45 | C G T A C A T G A G T C G A C T T G C A C G T A A C G T A C G T C T G A T C A G | Lhx1(Homeobox)/EmbryoCarcinoma-Lhx1-ChIP-Seq(GSE70957)/Homer | 1e-61 | -1.411e+02 | 0.0000 | 2386.0 | 24.69% | 6988.6 | 17.94% | motif file (matrix) | svg |
| 46 | C G T A C G T A C G T A G C A T G C A T T A C G G T A C G A C T A C T G C G T A A T C G A C G T G T A C C G T A A G C T | Bach1(bZIP)/K562-Bach1-ChIP-Seq(GSE31477)/Homer | 1e-45 | -1.046e+02 | 0.0000 | 211.0 | 2.18% | 266.5 | 0.68% | motif file (matrix) | svg |
| 47 | G T A C C G T A C G T A T A C G G C A T G T A C C G T A C A T G A G T C C G T A C G T A C G A T G C A T G C A T G A C T | MafF(bZIP)/HepG2-MafF-ChIP-Seq(GSE31477)/Homer | 1e-44 | -1.033e+02 | 0.0000 | 723.0 | 7.48% | 1666.3 | 4.28% | motif file (matrix) | svg |
| 48 | T G C A T C G A T C G A A C G T C A T G C G T A A G T C T C A G A C G T G T A C C G T A A G C T | CREB5(bZIP)/LNCaP-CREB5.V5-ChIP-Seq(GSE137775)/Homer | 1e-44 | -1.033e+02 | 0.0000 | 793.0 | 8.21% | 1882.3 | 4.83% | motif file (matrix) | svg |
| 49 | T A C G T C G A C A G T A C T G G C T A A T G C C G A T G T A C C G T A A C T G T A G C C G T A | NF-E2(bZIP)/K562-NFE2-ChIP-Seq(GSE31477)/Homer | 1e-42 | -9.728e+01 | 0.0000 | 212.0 | 2.19% | 283.0 | 0.73% | motif file (matrix) | svg |
| 50 | G C T A C G T A A G T C A C G T T C G A T A C G A C T G A G C T A G T C T C G A | RORgt(NR)/EL4-RORgt.Flag-ChIP-Seq(GSE56019)/Homer | 1e-41 | -9.512e+01 | 0.0000 | 341.0 | 3.53% | 602.8 | 1.55% | motif file (matrix) | svg |
| 51 | G C T A C G T A A G T C A C G T T C G A T A C G A C T G A G C T A G T C T C G A | RORgt(NR)/EL4-RORgt.Flag-ChIP-Seq(GSE56019)/Homer | 1e-41 | -9.512e+01 | 0.0000 | 341.0 | 3.53% | 602.8 | 1.55% | motif file (matrix) | svg |
| 52 | C G T A C G T A C G T A A G T C A G C T C T G A A C T G A C T G A G C T A G T C C G T A C T A G C T A G C T A G T G C A | RORa(NR)/Liver-Rora-ChIP-Seq(GSE101115)/Homer | 1e-41 | -9.499e+01 | 0.0000 | 377.0 | 3.90% | 698.5 | 1.79% | motif file (matrix) | svg |
| 53 | C G T A G A C T C G A T A T C G G T A C G C A T C A T G C G T A T A C G G C A T G T A C C G T A C A T G A T G C G C T A C T A G G C A T G C A T G C A T G A C T | MafB(bZIP)/BMM-Mafb-ChIP-Seq(GSE75722)/Homer | 1e-40 | -9.426e+01 | 0.0000 | 1088.0 | 11.26% | 2888.3 | 7.41% | motif file (matrix) | svg |
| 54 | C G T A C G T A C G T A A G T C A G C T C T G A A C T G C T A G A G C T A G T C C G T A T C A G | RORg(NR)/Liver-Rorc-ChIP-Seq(GSE101115)/Homer | 1e-39 | -9.167e+01 | 0.0000 | 270.0 | 2.79% | 431.5 | 1.11% | motif file (matrix) | svg |
| 55 | T C G A G C A T A C T G C T G A A G T C T C A G G A C T G T A C C G T A A G C T A G T C G A T C | c-Jun-CRE(bZIP)/K562-cJun-ChIP-Seq(GSE31477)/Homer | 1e-39 | -9.150e+01 | 0.0000 | 635.0 | 6.57% | 1458.8 | 3.74% | motif file (matrix) | svg |
| 56 | A T G C T C A G T C G A G C A T A C T G C G T A A G T C T C A G G A C T T G A C C G T A A G C T | Atf2(bZIP)/3T3L1-Atf2-ChIP-Seq(GSE56872)/Homer | 1e-39 | -9.022e+01 | 0.0000 | 671.0 | 6.94% | 1575.4 | 4.04% | motif file (matrix) | svg |
| 57 | T A G C C T A G T C G A G A C T A C T G C T G A A G T C T C A G G C A T T G A C C T G A A G C T | Atf7(bZIP)/3T3L1-Atf7-ChIP-Seq(GSE56872)/Homer | 1e-38 | -8.979e+01 | 0.0000 | 902.0 | 9.33% | 2309.4 | 5.93% | motif file (matrix) | svg |
| 58 | T C G A T A G C G T C A A C T G A C T G C G T A C G T A C T A G A G C T T C A G | ERG(ETS)/VCaP-ERG-ChIP-Seq(GSE14097)/Homer | 1e-36 | -8.316e+01 | 0.0000 | 2754.0 | 28.50% | 8943.3 | 22.96% | motif file (matrix) | svg |
| 59 | A T G C A G T C C T G A A G T C C G A T A C G T A G T C A G T C A C G T A T C G G A C T A C G T | Etv2(ETS)/ES-ER71-ChIP-Seq(GSE59402)/Homer | 1e-33 | -7.688e+01 | 0.0000 | 1727.0 | 17.87% | 5252.5 | 13.48% | motif file (matrix) | svg |
| 60 | C T A G T A C G G A T C G T A C G C T A A G C T A G C T G T C A T C G A T A G C | Nanog(Homeobox)/mES-Nanog-ChIP-Seq(GSE11724)/Homer | 1e-33 | -7.630e+01 | 0.0000 | 6353.0 | 65.75% | 23287.1 | 59.77% | motif file (matrix) | svg |
| 61 | A G T C C T G A A G T C C G A T C A G T G A T C A T G C A C T G A T C G G A C T | Fli1(ETS)/CD8-FLI-ChIP-Seq(GSE20898)/Homer | 1e-32 | -7.419e+01 | 0.0000 | 1904.0 | 19.70% | 5919.7 | 15.19% | motif file (matrix) | svg |
| 62 | T C A G T A G C G A C T C A T G C T G A A T C G G C A T G T A C C G T A A C T G T A G C T G C A | MafK(bZIP)/C2C12-MafK-ChIP-Seq(GSE36030)/Homer | 1e-32 | -7.372e+01 | 0.0000 | 606.0 | 6.27% | 1465.8 | 3.76% | motif file (matrix) | svg |
| 63 | A G C T C A T G G C A T G A T C T G C A C T A G G A T C A C G T | Tgif2(Homeobox)/mES-Tgif2-ChIP-Seq(GSE55404)/Homer | 1e-31 | -7.296e+01 | 0.0000 | 5152.0 | 53.32% | 18433.6 | 47.32% | motif file (matrix) | svg |
| 64 | T A C G T C G A G A C T A C T G C T G A A G T C T C A G G A C T T G A C C T G A | Atf1(bZIP)/K562-ATF1-ChIP-Seq(GSE31477)/Homer | 1e-31 | -7.282e+01 | 0.0000 | 1174.0 | 12.15% | 3349.4 | 8.60% | motif file (matrix) | svg |
| 65 | G T C A G C A T A C T G G T A C G A C T A C T G G C T A A T C G C A G T G T A C C G T A A G C T | Nrf2(bZIP)/Lymphoblast-Nrf2-ChIP-Seq(GSE37589)/Homer | 1e-29 | -6.907e+01 | 0.0000 | 165.0 | 1.71% | 234.5 | 0.60% | motif file (matrix) | svg |
| 66 | G A C T G C A T C T A G C G A T G A T C T C G A C A T G G A T C | Tgif1(Homeobox)/mES-Tgif1-ChIP-Seq(GSE55404)/Homer | 1e-28 | -6.600e+01 | 0.0000 | 4883.0 | 50.53% | 17472.2 | 44.85% | motif file (matrix) | svg |
| 67 | A G T C C G A T C T G A C G T A A C G T C A G T T C A G T G A C | Isl1(Homeobox)/Neuron-Isl1-ChIP-Seq(GSE31456)/Homer | 1e-28 | -6.458e+01 | 0.0000 | 3380.0 | 34.98% | 11589.1 | 29.75% | motif file (matrix) | svg |
| 68 | T C G A T A G C T G C A A C T G A C T G C G T A C G T A C T A G G A C T T A C G | ETS1(ETS)/Jurkat-ETS1-ChIP-Seq(GSE17954)/Homer | 1e-26 | -6.009e+01 | 0.0000 | 1846.0 | 19.10% | 5878.8 | 15.09% | motif file (matrix) | svg |
| 69 | T C G A A C G T A C T G C T G A A G T C T C A G A G C T G T A C C G T A A G C T G A T C T C G A | JunD(bZIP)/K562-JunD-ChIP-Seq/Homer | 1e-25 | -5.781e+01 | 0.0000 | 230.0 | 2.38% | 428.4 | 1.10% | motif file (matrix) | svg |
| 70 | A G C T A C G T A C T G A T G C A G T C C G T A C T G A T A C G | NF1-halfsite(CTF)/LNCaP-NF1-ChIP-Seq(Unpublished)/Homer | 1e-24 | -5.708e+01 | 0.0000 | 2832.0 | 29.31% | 9609.1 | 24.67% | motif file (matrix) | svg |
| 71 | T C G A A C G T A C G T C T G A G A T C T C A G G A C T G T C A C G T A A G C T G T C A C T A G A G C T A C G T T C G A | NFIL3(bZIP)/HepG2-NFIL3-ChIP-Seq(Encode)/Homer | 1e-23 | -5.360e+01 | 0.0000 | 1101.0 | 11.39% | 3279.5 | 8.42% | motif file (matrix) | svg |
| 72 | G A C T C G A T C T G A G T C A G A C T C G A T T C G A C G T A G C T A G C T A T G A C G T A C C G T A A C T G T G C A C G A T A C T G A C G T | Pitx1:Ebox(Homeobox,bHLH)/Hindlimb-Pitx1-ChIP-Seq(GSE41591)/Homer | 1e-22 | -5.069e+01 | 0.0000 | 447.0 | 4.63% | 1103.9 | 2.83% | motif file (matrix) | svg |
| 73 | C T G A T A G C T G A C T C A G C T A G G T C A C G T A T C A G A G C T T C A G | ETV4(ETS)/HepG2-ETV4-ChIP-Seq(ENCODE)/Homer | 1e-21 | -5.026e+01 | 0.0000 | 1779.0 | 18.41% | 5763.9 | 14.80% | motif file (matrix) | svg |
| 74 | T C A G T A C G T A G C A C G T A C T G C G A T A G T C C G T A T A C G A G T C | Meis1(Homeobox)/MastCells-Meis1-ChIP-Seq(GSE48085)/Homer | 1e-19 | -4.531e+01 | 0.0000 | 2836.0 | 29.35% | 9825.4 | 25.22% | motif file (matrix) | svg |
| 75 | T C A G A G C T A T G C C G T A A G C T T C A G C A G T A C T G C T G A A G T C | MITF(bHLH)/MastCells-MITF-ChIP-Seq(GSE48085)/Homer | 1e-18 | -4.206e+01 | 0.0000 | 1524.0 | 15.77% | 4941.2 | 12.68% | motif file (matrix) | svg |
| 76 | T C G A C T G A T A G C T G A C T C A G T C A G C G T A C G T A T C A G A G C T | ETV1(ETS)/GIST48-ETV1-ChIP-Seq(GSE22441)/Homer | 1e-18 | -4.186e+01 | 0.0000 | 2278.0 | 23.57% | 7759.8 | 19.92% | motif file (matrix) | svg |
| 77 | T C A G A C G T A G T C T C G A A G T C T C A G G C A T C T A G C T A G A G C T | Usf2(bHLH)/C2C12-Usf2-ChIP-Seq(GSE36030)/Homer | 1e-17 | -4.122e+01 | 0.0000 | 559.0 | 5.78% | 1534.1 | 3.94% | motif file (matrix) | svg |
| 78 | T C G A A G C T A C G T A C G T A G T C A G T C A C G T A T C G G A C T A T C G | EWS:ERG-fusion(ETS)/CADO\_ES1-EWS:ERG-ChIP-Seq(SRA014231)/Homer | 1e-17 | -4.103e+01 | 0.0000 | 1342.0 | 13.89% | 4290.4 | 11.01% | motif file (matrix) | svg |
| 79 | T G C A A G C T C A T G C G T A A G C T A C T G G A T C G T C A C G T A A G C T | Atf4(bZIP)/MEF-Atf4-ChIP-Seq(GSE35681)/Homer | 1e-17 | -4.042e+01 | 0.0000 | 524.0 | 5.42% | 1424.4 | 3.66% | motif file (matrix) | svg |
| 80 | A T G C A T C G T A C G A G C T A T C G C T G A A G T C C T A G A G C T A T G C C T G A A T G C | CRE(bZIP)/Promoter/Homer | 1e-17 | -3.983e+01 | 0.0000 | 367.0 | 3.80% | 918.8 | 2.36% | motif file (matrix) | svg |
| 81 | T G C A C T G A A T G C G T C A A C T G A C T G C G T A C G T A C T A G A G C T | Ets1-distal(ETS)/CD4+-PolII-ChIP-Seq(Barski\_et\_al.)/Homer | 1e-16 | -3.885e+01 | 0.0000 | 592.0 | 6.13% | 1665.7 | 4.28% | motif file (matrix) | svg |
| 82 | C A T G G A C T T A C G G T C A G T A C G A T C G A C T A G C T A T C G T C G A T A C G T A G C | ERRg(NR)/Kidney-ESRRG-ChIP-Seq(GSE104905)/Homer | 1e-16 | -3.789e+01 | 0.0000 | 1338.0 | 13.85% | 4319.4 | 11.09% | motif file (matrix) | svg |
| 83 | A G T C A C G T A C T G A G C T A C G T A C G T G T C A A G T C | Foxo1(Forkhead)/RAW-Foxo1-ChIP-Seq(Fan\_et\_al.)/Homer | 1e-16 | -3.762e+01 | 0.0000 | 3084.0 | 31.92% | 10933.0 | 28.06% | motif file (matrix) | svg |
| 84 | C T A G T C G A C G A T C T A G G C A T C A G T C T A G G A T C C G T A G T C A | CEBP:AP1(bZIP)/ThioMac-CEBPb-ChIP-Seq(GSE21512)/Homer | 1e-16 | -3.754e+01 | 0.0000 | 1295.0 | 13.40% | 4167.5 | 10.70% | motif file (matrix) | svg |
| 85 | T C G A A C T G A C T G C G T A C G T A T C G A A G T C C T G A A T C G G T A C G C A T C A T G | ETS:E-box(ETS,bHLH)/HPC7-Scl-ChIP-Seq(GSE22178)/Homer | 1e-15 | -3.678e+01 | 0.0000 | 212.0 | 2.19% | 460.4 | 1.18% | motif file (matrix) | svg |
| 86 | T G C A C G T A G T C A A G C T A G T C G C T A T A G C C G A T C T A G G A T C | Gfi1b(Zf)/HPC7-Gfi1b-ChIP-Seq(GSE22178)/Homer | 1e-15 | -3.675e+01 | 0.0000 | 1236.0 | 12.79% | 3963.9 | 10.17% | motif file (matrix) | svg |
| 87 | T G C A C T G A A G T C G T C A A C T G A C T G C G T A C G T A C T G A A G C T | EWS:FLI1-fusion(ETS)/SK\_N\_MC-EWS:FLI1-ChIP-Seq(SRA014231)/Homer | 1e-15 | -3.497e+01 | 0.0000 | 1041.0 | 10.77% | 3281.0 | 8.42% | motif file (matrix) | svg |
| 88 | T C A G G A C T C A G T C T G A A G C T C T A G G A C T T G C A C T G A A G T C | HLF(bZIP)/HSC-HLF.Flag-ChIP-Seq(GSE69817)/Homer | 1e-14 | -3.394e+01 | 0.0000 | 1379.0 | 14.27% | 4528.4 | 11.62% | motif file (matrix) | svg |
| 89 | T G A C G C T A T G A C C G T A T C A G G A T C C G T A C A T G C A T G C T A G C T A G C T A G | Unknown-ESC-element(?)/mES-Nanog-ChIP-Seq(GSE11724)/Homer | 1e-14 | -3.360e+01 | 0.0000 | 991.0 | 10.26% | 3119.2 | 8.01% | motif file (matrix) | svg |
| 90 | C T A G G C A T G A T C C G T A A G T C T C A G G A C T C T A G | CLOCK(bHLH)/Liver-Clock-ChIP-Seq(GSE39860)/Homer | 1e-14 | -3.290e+01 | 0.0000 | 858.0 | 8.88% | 2652.2 | 6.81% | motif file (matrix) | svg |
| 91 | C A T G G T A C C G T A A G T C C T A G A C G T A C T G G T A C A G T C A G C T | bHLHE40(bHLH)/HepG2-BHLHE40-ChIP-Seq(GSE31477)/Homer | 1e-14 | -3.247e+01 | 0.0000 | 469.0 | 4.85% | 1306.3 | 3.35% | motif file (matrix) | svg |
| 92 | G A C T G T A C T G C A A C G T G A T C G C T A T C G A A C G T A G T C C G T A | Pdx1(Homeobox)/Islet-Pdx1-ChIP-Seq(SRA008281)/Homer | 1e-14 | -3.243e+01 | 0.0000 | 1702.0 | 17.61% | 5754.2 | 14.77% | motif file (matrix) | svg |
| 93 | C A T G T A C G T A G C G A T C G A T C A T G C G T A C G A C T T C A G A T G C C G A T A T C G C A G T A C T G G T A C | Zic3(Zf)/mES-Zic3-ChIP-Seq(GSE37889)/Homer | 1e-13 | -3.218e+01 | 0.0000 | 890.0 | 9.21% | 2775.8 | 7.13% | motif file (matrix) | svg |
| 94 | T C G A G C A T A C G T C T A G G T A C T C G A G C A T T G A C T C G A A C G T | Chop(bZIP)/MEF-Chop-ChIP-Seq(GSE35681)/Homer | 1e-13 | -3.165e+01 | 0.0000 | 407.0 | 4.21% | 1107.0 | 2.84% | motif file (matrix) | svg |
| 95 | G A C T C T A G A T G C A G T C G T C A T A C G A T G C A T C G | HIC1(Zf)/Treg-ZBTB29-ChIP-Seq(GSE99889)/Homer | 1e-12 | -2.978e+01 | 0.0000 | 3054.0 | 31.61% | 10989.9 | 28.21% | motif file (matrix) | svg |
| 96 | T C G A T C G A T A G C G T A C T C A G T A C G C G T A C G T A T C A G A G C T | GABPA(ETS)/Jurkat-GABPa-ChIP-Seq(GSE17954)/Homer | 1e-12 | -2.894e+01 | 0.0000 | 1439.0 | 14.89% | 4832.3 | 12.40% | motif file (matrix) | svg |
| 97 | T A C G T C A G A G C T A T G C C G T A A G T C T C A G A C G T A C T G T C G A | USF1(bHLH)/GM12878-Usf1-ChIP-Seq(GSE32465)/Homer | 1e-12 | -2.856e+01 | 0.0000 | 693.0 | 7.17% | 2119.2 | 5.44% | motif file (matrix) | svg |
| 98 | C A T G A G C T T A C G G T C A G T A C T A G C A G C T G A C T A T C G T C G A | Esrrb(NR)/mES-Esrrb-ChIP-Seq(GSE11431)/Homer | 1e-11 | -2.722e+01 | 0.0000 | 1070.0 | 11.07% | 3496.0 | 8.97% | motif file (matrix) | svg |
| 99 | T C G A T A G C T G A C C T G A A G T C A C T G G A C T C A T G | c-Myc(bHLH)/LNCAP-cMyc-ChIP-Seq(Unpublished)/Homer | 1e-11 | -2.711e+01 | 0.0000 | 595.0 | 6.16% | 1791.7 | 4.60% | motif file (matrix) | svg |
| 100 | T C A G A G C T A T G C C G T A A G T C T C A G A C G T A T C G T C G A A G T C G A T C T G A C | TFE3(bHLH)/MEF-TFE3-ChIP-Seq(GSE75757)/Homer | 1e-11 | -2.702e+01 | 0.0000 | 156.0 | 1.61% | 341.6 | 0.88% | motif file (matrix) | svg |
| 101 | T A C G A T G C G A C T A C T G A G C T A G T C G T C A T G C A A C G T A G T C G C T A T G C A | Pknox1(Homeobox)/ES-Prep1-ChIP-Seq(GSE63282)/Homer | 1e-11 | -2.674e+01 | 0.0000 | 424.0 | 4.39% | 1205.1 | 3.09% | motif file (matrix) | svg |
| 102 | A G T C G C A T C G T A C G T A G T A C A C G T A C T G G A T C G A T C T C G A | BMYB(HTH)/Hela-BMYB-ChIP-Seq(GSE27030)/Homer | 1e-11 | -2.621e+01 | 0.0000 | 2380.0 | 24.63% | 8460.7 | 21.72% | motif file (matrix) | svg |
| 103 | C G T A C G T A C G T A G C A T G C A T A C T G G T A C G A C T C T A G G C T A T A C G G A C T T G A C C G T A A G C T | NFE2L2(bZIP)/HepG2-NFE2L2-ChIP-Seq(Encode)/Homer | 1e-11 | -2.611e+01 | 0.0000 | 170.0 | 1.76% | 387.2 | 0.99% | motif file (matrix) | svg |
| 104 | T A G C T A G C G A C T C T A G A G C T A G T C G T C A T G C A A C G T A T G C G C T A T G C A | Pbx3(Homeobox)/GM12878-PBX3-ChIP-Seq(GSE32465)/Homer | 1e-11 | -2.539e+01 | 0.0000 | 456.0 | 4.72% | 1327.3 | 3.41% | motif file (matrix) | svg |
| 105 | C A T G A C T G C T A G T C G A T C G A T C G A T C G A T C A G T C A G T C A G T G A C T G A C C G T A A C T G T G C A C G A T A C T G | RBPJ:Ebox(?,bHLH)/Panc1-Rbpj1-ChIP-Seq(GSE47459)/Homer | 1e-10 | -2.519e+01 | 0.0000 | 558.0 | 5.77% | 1683.2 | 4.32% | motif file (matrix) | svg |
| 106 | C A T G C T A G A G C T G A C T C A T G A G T C G A T C G C T A C G A T C T A G T C A G G T A C C T G A T C G A | X-box(HTH)/NPC-H3K4me1-ChIP-Seq(GSE16256)/Homer | 1e-10 | -2.445e+01 | 0.0000 | 199.0 | 2.06% | 485.0 | 1.25% | motif file (matrix) | svg |
| 107 | C T A G A T G C A T G C C G A T A C T G G A C T A T G C G C T A T G A C A G C T T A G C G C T A | PBX1(Homeobox)/MCF7-PBX1-ChIP-Seq(GSE28007)/Homer | 1e-10 | -2.415e+01 | 0.0000 | 181.0 | 1.87% | 431.2 | 1.11% | motif file (matrix) | svg |
| 108 | C T G A A G T C C G A T A G C T A T G C G T A C A C G T A T C G C A G T G C A T | Elf4(ETS)/BMDM-Elf4-ChIP-Seq(GSE88699)/Homer | 1e-10 | -2.380e+01 | 0.0000 | 1647.0 | 17.04% | 5711.5 | 14.66% | motif file (matrix) | svg |
| 109 | G C A T C G T A C G A T A C T G A G T C G C T A C T G A C G T A C A G T A C T G C G T A T C A G | Oct6(POU,Homeobox)/NPC-Pou3f1-ChIP-Seq(GSE35496)/Homer | 1e-10 | -2.364e+01 | 0.0000 | 845.0 | 8.74% | 2729.4 | 7.01% | motif file (matrix) | svg |
| 110 | C T A G T A C G G A T C G T C A T G C A A C G T T G C A G C T A T C G A T G C A | Hoxa9(Homeobox)/ChickenMSG-Hoxa9.Flag-ChIP-Seq(GSE86088)/Homer | 1e-10 | -2.343e+01 | 0.0000 | 4375.0 | 45.28% | 16376.3 | 42.04% | motif file (matrix) | svg |
| 111 | C G T A T A G C T A G C T G C A A C T G C T A G C G T A C G T A T C A G G A C T | EHF(ETS)/LoVo-EHF-ChIP-Seq(GSE49402)/Homer | 1e-10 | -2.323e+01 | 0.0000 | 2106.0 | 21.79% | 7475.5 | 19.19% | motif file (matrix) | svg |
| 112 | A G T C G A C T C A G T A C T G C T A G T G A C G C T A A T G C G C A T A T C G C G A T A C T G G A T C G T A C G T C A C T G A | NF1(CTF)/LNCAP-NF1-ChIP-Seq(Unpublished)/Homer | 1e-9 | -2.239e+01 | 0.0000 | 579.0 | 5.99% | 1790.9 | 4.60% | motif file (matrix) | svg |
| 113 | G A C T C G T A A C G T A C T G A G T C C G T A C T G A C G T A C A G T A C G T G T C A T C A G | Brn1(POU,Homeobox)/NPC-Brn1-ChIP-Seq(GSE35496)/Homer | 1e-9 | -2.087e+01 | 0.0000 | 622.0 | 6.44% | 1963.9 | 5.04% | motif file (matrix) | svg |
| 114 | C G A T C T A G A C G T G T C A C G T A C G T A A G T C C G T A | Foxo3(Forkhead)/U2OS-Foxo3-ChIP-Seq(E-MTAB-2701)/Homer | 1e-8 | -2.070e+01 | 0.0000 | 1238.0 | 12.81% | 4232.3 | 10.86% | motif file (matrix) | svg |
| 115 | T A G C T C A G C A T G G C A T A G C T C G A T A T G C C G T A C G T A G T C A | CHR(?)/Hela-CellCycle-Expression/Homer | 1e-8 | -2.038e+01 | 0.0000 | 1076.0 | 11.14% | 3634.0 | 9.33% | motif file (matrix) | svg |
| 116 | C G T A A C G T A G C T C G A T C T A G G T A C C G T A A G C T C G T A G C T A | Oct4(POU,Homeobox)/mES-Oct4-ChIP-Seq(GSE11431)/Homer | 1e-8 | -2.029e+01 | 0.0000 | 884.0 | 9.15% | 2924.6 | 7.51% | motif file (matrix) | svg |
| 117 | G A T C C T G A A G T C C G A T C G A T G A T C A G T C A C T G A T C G A G C T | Elk4(ETS)/Hela-Elk4-ChIP-Seq(GSE31477)/Homer | 1e-8 | -1.997e+01 | 0.0000 | 780.0 | 8.07% | 2548.6 | 6.54% | motif file (matrix) | svg |
| 118 | A T G C A G T C G C T A C G A T C G T A G C A T G C T A C G A T C T A G C A T G T G A C G T C A | CArG(MADS)/PUER-Srf-ChIP-Seq(Sullivan\_et\_al.)/Homer | 1e-8 | -1.990e+01 | 0.0000 | 515.0 | 5.33% | 1594.4 | 4.09% | motif file (matrix) | svg |
| 119 | C T A G T A C G G A T C G T A C C T G A A G C T T G C A G C T A C G T A G C A T G A T C G C T A | Hoxc9(Homeobox)/Ainv15-Hoxc9-ChIP-Seq(GSE21812)/Homer | 1e-8 | -1.965e+01 | 0.0000 | 874.0 | 9.04% | 2898.0 | 7.44% | motif file (matrix) | svg |
| 120 | A G T C C G A T A C T G A T C G T G A C G C T A C A T G A T C G T G A C C G A T A C T G T A G C G T A C G T C A | Tlx?(NR)/NPC-H3K4me1-ChIP-Seq(GSE16256)/Homer | 1e-8 | -1.938e+01 | 0.0000 | 710.0 | 7.35% | 2302.7 | 5.91% | motif file (matrix) | svg |
| 121 | T G C A G C A T C G A T C G T A C A G T A C T G G T A C C G T A C T G A A G C T G T C A A C T G C T A G G T C A C G A T A C T G G T A C T G C A C G T A A G C T | CEBP:CEBP(bZIP)/MEF-Chop-ChIP-Seq(GSE35681)/Homer | 1e-8 | -1.855e+01 | 0.0000 | 276.0 | 2.86% | 779.5 | 2.00% | motif file (matrix) | svg |
| 122 | G A T C T C G A A G T C C G A T C G A T A G T C A T G C A C T G A T C G G A C T | Elk1(ETS)/Hela-Elk1-ChIP-Seq(GSE31477)/Homer | 1e-8 | -1.854e+01 | 0.0000 | 759.0 | 7.85% | 2494.7 | 6.40% | motif file (matrix) | svg |
| 123 | T C A G A T C G G A C T A C T G G A C T C A G T C T A G C G T A G T A C C G T A C T A G A T C G | Tbx20(T-box)/Heart-Tbx20-ChIP-Seq(GSE29636)/Homer | 1e-7 | -1.835e+01 | 0.0000 | 401.0 | 4.15% | 1211.1 | 3.11% | motif file (matrix) | svg |
| 124 | A T G C T C G A T A C G A C G T A T G C A G T C A C G T A G T C A G T C G A T C | Znf263(Zf)/K562-Znf263-ChIP-Seq(GSE31477)/Homer | 1e-7 | -1.773e+01 | 0.0000 | 2558.0 | 26.47% | 9372.0 | 24.06% | motif file (matrix) | svg |
| 125 | T G A C C T A G A C T G T A G C C G A T A C T G A T G C C A T G A T C G A T C G A T C G T A G C C T G A T A G C G C T A A C T G C G T A A G C T C G T A C T G A | GATA:SCL(Zf,bHLH)/Ter119-SCL-ChIP-Seq(GSE18720)/Homer | 1e-7 | -1.752e+01 | 0.0000 | 218.0 | 2.26% | 594.8 | 1.53% | motif file (matrix) | svg |
| 126 | A T G C A T G C A T C G T A C G A G C T A G T C G C T A A G T C T C A G G A C T A C T G T C G A | E-box(bHLH)/Promoter/Homer | 1e-7 | -1.648e+01 | 0.0000 | 108.0 | 1.12% | 250.6 | 0.64% | motif file (matrix) | svg |
| 127 | T A G C G T A C A G T C G T A C C G A T A G T C A G T C A G T C A G T C A G T C C G T A G A T C | Zfp281(Zf)/ES-Zfp281-ChIP-Seq(GSE81042)/Homer | 1e-7 | -1.641e+01 | 0.0000 | 319.0 | 3.30% | 947.2 | 2.43% | motif file (matrix) | svg |
| 128 | C T A G A G C T G A C T C A T G A G T C A G T C G T C A C A G T C T A G T C A G G T A C C T G A T C G A G A T C T G A C | Rfx2(HTH)/LoVo-RFX2-ChIP-Seq(GSE49402)/Homer | 1e-7 | -1.628e+01 | 0.0000 | 150.0 | 1.55% | 382.9 | 0.98% | motif file (matrix) | svg |
| 129 | G A C T C T A G C T A G C T A G A C T G T C G A C T G A C T A G C T A G C T A G G T A C G T C A | ZNF467(Zf)/HEK293-ZNF467.GFP-ChIP-Seq(GSE58341)/Homer | 1e-7 | -1.623e+01 | 0.0000 | 1147.0 | 11.87% | 3982.8 | 10.22% | motif file (matrix) | svg |
| 130 | C T G A C G A T C T A G T C A G G A T C C T G A T C A G G A T C C T G A A C T G A G T C G C T A A C G T A G T C G C A T | PRDM9(Zf)/Testis-DMC1-ChIP-Seq(GSE35498)/Homer | 1e-6 | -1.591e+01 | 0.0000 | 732.0 | 7.58% | 2439.9 | 6.26% | motif file (matrix) | svg |
| 131 | T C A G A G C T A C G T A C G T G T A C G A T C C G T A C T A G C A T G G T C A C G T A T C G A | STAT4(Stat)/CD4-Stat4-ChIP-Seq(GSE22104)/Homer | 1e-6 | -1.563e+01 | 0.0000 | 1537.0 | 15.91% | 5479.4 | 14.06% | motif file (matrix) | svg |
| 132 | T A C G T A G C G C T A C G A T C T A G A C G T C A G T C A G T G C T A A G T C G T C A G C A T | FOXK2(Forkhead)/U2OS-FOXK2-ChIP-Seq(E-MTAB-2204)/Homer | 1e-6 | -1.551e+01 | 0.0000 | 988.0 | 10.22% | 3399.5 | 8.73% | motif file (matrix) | svg |
| 133 | C T A G T A C G G A C T T G C A T G C A C G A T T A C G C T G A T C G A C T G A | Hoxa10(Homeobox)/ChickenMSG-Hoxa10.Flag-ChIP-Seq(GSE86088)/Homer | 1e-6 | -1.519e+01 | 0.0000 | 979.0 | 10.13% | 3372.6 | 8.66% | motif file (matrix) | svg |
| 134 | A C T G G T C A A C G T G C A T C G A T T C A G G T A C G T C A A C G T C T G A | Oct11(POU,Homeobox)/NCIH1048-POU2F3-ChIP-seq(GSE115123)/Homer | 1e-6 | -1.442e+01 | 0.0000 | 598.0 | 6.19% | 1972.2 | 5.06% | motif file (matrix) | svg |
| 135 | C T G A C G A T C T A G C G T A A G C T C G A T C A G T C T G A G A C T C T A G C T A G A T G C | PBX2(Homeobox)/K562-PBX2-ChIP-Seq(Encode)/Homer | 1e-6 | -1.425e+01 | 0.0000 | 1384.0 | 14.32% | 4929.1 | 12.65% | motif file (matrix) | svg |
| 136 | C T G A A G C T A C G T A C G T A G T C G A C T G A C T C T G A C T G A C T A G C G T A C G T A | STAT6(Stat)/CD4-Stat6-ChIP-Seq(GSE22104)/Homer | 1e-6 | -1.409e+01 | 0.0000 | 871.0 | 9.01% | 2990.2 | 7.68% | motif file (matrix) | svg |
| 137 | C G T A T G A C T A G C T G C A A C T G A C T G C G T A C G T A T C A G G A C T | ELF3(ETS)/PDAC-ELF3-ChIP-Seq(GSE64557)/Homer | 1e-6 | -1.393e+01 | 0.0000 | 1218.0 | 12.60% | 4304.0 | 11.05% | motif file (matrix) | svg |
| 138 | T G A C G T C A A G T C G C T A C T A G A G T C C T G A C A T G A C T G C T A G T A C G T C A G | Zic2(Zf)/ESC-Zic2-ChIP-Seq(SRP197560)/Homer | 1e-5 | -1.356e+01 | 0.0000 | 628.0 | 6.50% | 2097.9 | 5.38% | motif file (matrix) | svg |
| 139 | G A C T C A G T G A T C G A T C A C G T G A T C C T G A T A C G C G T A G T C A | STAT6(Stat)/Macrophage-Stat6-ChIP-Seq(GSE38377)/Homer | 1e-5 | -1.333e+01 | 0.0000 | 891.0 | 9.22% | 3081.3 | 7.91% | motif file (matrix) | svg |
| 140 | T G A C C T G A C T A G C T G A C G T A A G T C C T G A A C G T G C A T T A G C G C A T A T C G G A C T G A C T G A T C | GRE(NR),IR3/RAW264.7-GRE-ChIP-Seq(Unpublished)/Homer | 1e-5 | -1.295e+01 | 0.0000 | 380.0 | 3.93% | 1204.4 | 3.09% | motif file (matrix) | svg |
| 141 | A G T C A T C G C T A G A G C T G A C T C T A G A G T C A G T C G C T A C A G T T C A G T C A G G A T C C T G A T C G A G A T C | RFX(HTH)/K562-RFX3-ChIP-Seq(SRA012198)/Homer | 1e-5 | -1.284e+01 | 0.0000 | 128.0 | 1.32% | 335.7 | 0.86% | motif file (matrix) | svg |
| 142 | C T G A A T G C C G T A A C G T A G T C A G T C A C G T A C T G A T C G G C A T | SPDEF(ETS)/VCaP-SPDEF-ChIP-Seq(SRA014231)/Homer | 1e-5 | -1.234e+01 | 0.0000 | 1654.0 | 17.12% | 6021.5 | 15.46% | motif file (matrix) | svg |
| 143 | T G A C G T A C C G T A A C T G T G A C C G A T A C T G A T C G A G C T T A C G T C G A T A G C G T A C C G T A A T C G T G A C G C A T A C T G A C T G A T G C | Twist(bHLH)/HMLE-TWIST1-ChIP-Seq(Chang\_et\_al)/Homer | 1e-5 | -1.229e+01 | 0.0000 | 243.0 | 2.51% | 728.1 | 1.87% | motif file (matrix) | svg |
| 144 | C G A T C T A G T C G A A G C T C G A T C T G A C G T A A G C T A C T G C T A G A T G C G A T C | Hoxb4(Homeobox)/ES-Hoxb4-ChIP-Seq(GSE34014)/Homer | 1e-5 | -1.222e+01 | 0.0000 | 342.0 | 3.54% | 1079.0 | 2.77% | motif file (matrix) | svg |
| 145 | A C T G T C A G A G C T G A C T C A T G A G T C A G T C G C T A C G A T C T A G T C A G G T A C C T G A T C G A | Rfx1(HTH)/NPC-H3K4me1-ChIP-Seq(GSE16256)/Homer | 1e-5 | -1.208e+01 | 0.0000 | 301.0 | 3.11% | 934.8 | 2.40% | motif file (matrix) | svg |
| 146 | A T G C G A T C G A C T A G C T C G A T C G A T G T C A C G A T T C G A A T C G T A G C T A G C | TATA-Box(TBP)/Promoter/Homer | 1e-5 | -1.189e+01 | 0.0000 | 2130.0 | 22.04% | 7886.5 | 20.24% | motif file (matrix) | svg |
| 147 | G T C A G C A T G C T A C A G T C T A G G A T C C G T A C T G A C G T A C G A T | Oct2(POU,Homeobox)/Bcell-Oct2-ChIP-Seq(GSE21512)/Homer | 1e-4 | -1.132e+01 | 0.0000 | 555.0 | 5.74% | 1868.2 | 4.80% | motif file (matrix) | svg |
| 148 | G C A T T C G A C T G A G A T C A G T C G A T C G T C A G T C A A C G T A G T C C G T A C T G A | Duxbl(Homeobox)/NIH3T3-Duxbl.HA-ChIP-Seq(GSE119782)/Homer | 1e-4 | -1.090e+01 | 0.0001 | 129.0 | 1.33% | 353.4 | 0.91% | motif file (matrix) | svg |
| 149 | T G A C C G T A C T G A A C T G A C T G G A C T G A T C T G C A G T A C T A C G | SF1(NR)/H295R-Nr5a1-ChIP-Seq(GSE44220)/Homer | 1e-4 | -1.059e+01 | 0.0001 | 830.0 | 8.59% | 2913.8 | 7.48% | motif file (matrix) | svg |
| 150 | T C G A T A G C G T C A A C T G C T A G C G T A C G A T A C T G A C G T A C T G A C T G A C G T | ETS:RUNX(ETS,Runt)/Jurkat-RUNX1-ChIP-Seq(GSE17954)/Homer | 1e-4 | -1.058e+01 | 0.0001 | 124.0 | 1.28% | 339.8 | 0.87% | motif file (matrix) | svg |
| 151 | C T G A T G C A T A G C T G A C T A C G T C A G C T G A G C T A T C A G G A C T | ELF1(ETS)/Jurkat-ELF1-ChIP-Seq(SRA014231)/Homer | 1e-4 | -1.048e+01 | 0.0001 | 686.0 | 7.10% | 2373.6 | 6.09% | motif file (matrix) | svg |
| 152 | A T G C C T G A A T C G T A C G A G T C C G A T T C A G C G A T C T A G A G C T G T C A G T C A C G T A A G T C C G T A T A C G C T G A | Fox:Ebox(Forkhead,bHLH)/Panc1-Foxa2-ChIP-Seq(GSE47459)/Homer | 1e-4 | -1.041e+01 | 0.0001 | 1633.0 | 16.90% | 6001.7 | 15.41% | motif file (matrix) | svg |
| 153 | C T G A C T G A C A T G A T C G A G C T A T C G G A C T C A T G C T G A G T C A | Tbr1(T-box)/Cortex-Tbr1-ChIP-Seq(GSE71384)/Homer | 1e-4 | -9.803e+00 | 0.0002 | 2023.0 | 20.94% | 7543.6 | 19.36% | motif file (matrix) | svg |
| 154 | A C T G C G T A A C T G A T G C T G A C G A T C A T C G T G C A A C T G A G T C | ZNF519(Zf)/HEK293-ZNF519.GFP-ChIP-Seq(GSE58341)/Homer | 1e-4 | -9.719e+00 | 0.0002 | 286.0 | 2.96% | 912.0 | 2.34% | motif file (matrix) | svg |
| 155 | C G T A A C G T A C G T A C G T A C G T A G T C A G T C C T G A A G C T A G C T | NFAT(RHD)/Jurkat-NFATC1-ChIP-Seq(Jolma\_et\_al.)/Homer | 1e-4 | -9.458e+00 | 0.0002 | 1417.0 | 14.66% | 5195.0 | 13.33% | motif file (matrix) | svg |
| 156 | A T G C G A C T A G C T A G C T A G T C G C T A C A G T C G A T G C T A A C G T A C T G G C T A T A G C G C A T T G A C | IRF:BATF(IRF:bZIP)/pDC-Irf8-ChIP-Seq(GSE66899)/Homer | 1e-3 | -8.797e+00 | 0.0004 | 217.0 | 2.25% | 678.9 | 1.74% | motif file (matrix) | svg |
| 157 | C A T G A C G T A G T C G A T C G A T C G A T C G C T A C T A G C T A G C T A G T C A G T C G A | EBF1(EBF)/Near-E2A-ChIP-Seq(GSE21512)/Homer | 1e-3 | -8.730e+00 | 0.0005 | 1294.0 | 13.39% | 4743.8 | 12.18% | motif file (matrix) | svg |
| 158 | T C A G G C T A T C A G C A G T T G A C G T C A A G T C A T C G T G C A G T A C C A G T G A T C | Npas4(bHLH)/Neuron-Npas4-ChIP-Seq(GSE127793)/Homer | 1e-3 | -8.718e+00 | 0.0005 | 990.0 | 10.25% | 3572.4 | 9.17% | motif file (matrix) | svg |
| 159 | C A G T T C A G A G C T G A C T A C G T A G T C G A T C G A C T C T G A A C T G G A T C C G T A C T G A A G T C G T A C | Rfx6(HTH)/Min6b1-Rfx6.HA-ChIP-Seq(GSE62844)/Homer | 1e-3 | -8.478e+00 | 0.0006 | 1605.0 | 16.61% | 5960.9 | 15.30% | motif file (matrix) | svg |
| 160 | G C T A A G C T T A C G G T C A C T G A C G A T C T G A G C A T C A G T A G T C | Brn2(POU,Homeobox)/NPC-Brn2-ChIP-Seq(GSE35496)/Homer | 1e-3 | -8.368e+00 | 0.0006 | 159.0 | 1.65% | 479.6 | 1.23% | motif file (matrix) | svg |
| 161 | A G C T A G T C A G T C A C G T C T A G A C G T A C G T A C G T C G T A A G T C G A T C C G T A | FOXP1(Forkhead)/H9-FOXP1-ChIP-Seq(GSE31006)/Homer | 1e-3 | -8.220e+00 | 0.0007 | 705.0 | 7.30% | 2498.0 | 6.41% | motif file (matrix) | svg |
| 162 | C G T A C T G A A C T G A G C T A G T C G A T C G A T C G C A T C T G A C T A G C T A G T A C G T C G A T G C A G C A T | EBF2(EBF)/BrownAdipose-EBF2-ChIP-Seq(GSE97114)/Homer | 1e-3 | -7.936e+00 | 0.0010 | 1215.0 | 12.57% | 4464.3 | 11.46% | motif file (matrix) | svg |
| 163 | C T A G A C T G C T A G T C A G T C A G T A C G C T A G A C T G | Maz(Zf)/HepG2-Maz-ChIP-Seq(GSE31477)/Homer | 1e-3 | -7.810e+00 | 0.0011 | 1432.0 | 14.82% | 5311.3 | 13.63% | motif file (matrix) | svg |
| 164 | A T G C G A C T A C G T C T A G A C G T A C G T A C G T C T G A G A T C G C T A A G C T C G T A | Foxa2(Forkhead)/Liver-Foxa2-ChIP-Seq(GSE25694)/Homer | 1e-3 | -7.774e+00 | 0.0011 | 1260.0 | 13.04% | 4644.7 | 11.92% | motif file (matrix) | svg |
| 165 | A G T C G T A C A G C T C T A G A G T C C G A T A C T G C G T A A C T G G T C A | Zic(Zf)/Cerebellum-ZIC1.2-ChIP-Seq(GSE60731)/Homer | 1e-3 | -7.748e+00 | 0.0012 | 1251.0 | 12.95% | 4610.4 | 11.83% | motif file (matrix) | svg |
| 166 | C G T A A C G T A C T G G T A C C G T A A C G T C G T A C G T A A C G T A C T G A G T C C G T A A C G T C T G A G C A T | OCT:OCT-short(POU,Homeobox)/NPC-OCT6-ChIP-Seq(GSE43916)/Homer | 1e-3 | -7.667e+00 | 0.0012 | 1170.0 | 12.11% | 4299.1 | 11.04% | motif file (matrix) | svg |
| 167 | A T G C T C G A A G T C A G C T A C G T G T A C A G T C G C T A C T A G C A T G G T C A C T G A T C A G A G T C | Stat3+il21(Stat)/CD4-Stat3-ChIP-Seq(GSE19198)/Homer | 1e-3 | -7.634e+00 | 0.0013 | 1081.0 | 11.19% | 3956.9 | 10.16% | motif file (matrix) | svg |
| 168 | T C G A G C A T A T G C C T G A A T G C T A G C A G T C G T A C T C G A A G C T | Srebp1a(bHLH)/HepG2-Srebp1a-ChIP-Seq(GSE31477)/Homer | 1e-3 | -7.530e+00 | 0.0014 | 276.0 | 2.86% | 909.7 | 2.34% | motif file (matrix) | svg |
| 169 | G C T A A G T C T A C G T G C A A T C G T C A G G C T A T C G A T C A G A G C T | ELF5(ETS)/T47D-ELF5-ChIP-Seq(GSE30407)/Homer | 1e-3 | -7.325e+00 | 0.0017 | 1161.0 | 12.01% | 4276.8 | 10.98% | motif file (matrix) | svg |
| 170 | A C G T G A C T T A G C C G T A C T G A C A T G C T A G G A C T G A T C C G T A | Nr5a2(NR)/Pancreas-LRH1-ChIP-Seq(GSE34295)/Homer | 1e-3 | -7.273e+00 | 0.0018 | 1176.0 | 12.17% | 4336.5 | 11.13% | motif file (matrix) | svg |
| 171 | C G T A C T G A C G T A C T A G T C G A C T A G A C T G C G T A C G T A T A C G A G C T A T C G | SpiB(ETS)/OCILY3-SPIB-ChIP-Seq(GSE56857)/Homer | 1e-3 | -7.066e+00 | 0.0022 | 349.0 | 3.61% | 1186.3 | 3.05% | motif file (matrix) | svg |
| 172 | A G C T T G A C C G A T C G A T C T A G A C G T C A G T C A G T G C T A A G T C | FOXK1(Forkhead)/HEK293-FOXK1-ChIP-Seq(GSE51673)/Homer | 1e-3 | -7.044e+00 | 0.0022 | 1622.0 | 16.79% | 6084.0 | 15.62% | motif file (matrix) | svg |
| 173 | C A T G T G A C C G T A A G T C T A C G G C A T A C T G G T C A A T G C A G T C | bHLHE41(bHLH)/proB-Bhlhe41-ChIP-Seq(GSE93764)/Homer | 1e-3 | -7.012e+00 | 0.0023 | 1470.0 | 15.21% | 5490.4 | 14.09% | motif file (matrix) | svg |
| 174 | C T G A C T G A C T A G T C G A C G T A A T G C C G T A A C T G C G T A A C G T C T G A C G A T A G C T C G T A A C G T A G T C C G A T T A C G G T C A G C A T | GATA(Zf),IR3/iTreg-Gata3-ChIP-Seq(GSE20898)/Homer | 1e-2 | -6.760e+00 | 0.0029 | 270.0 | 2.79% | 900.4 | 2.31% | motif file (matrix) | svg |
| 175 | G C T A G A C T G A C T T G C A C G T A A G T C C G T A T A G C G A T C G A C T | Eomes(T-box)/H9-Eomes-ChIP-Seq(GSE26097)/Homer | 1e-2 | -6.627e+00 | 0.0033 | 3092.0 | 32.00% | 11913.1 | 30.58% | motif file (matrix) | svg |
| 176 | C G A T C T A G C G T A G A C T C A G T C T A G C G T A A G C T C A T G C T A G | HOXA1(Homeobox)/mES-Hoxa1-ChIP-Seq(SRP084292)/Homer | 1e-2 | -6.419e+00 | 0.0041 | 436.0 | 4.51% | 1525.6 | 3.92% | motif file (matrix) | svg |
| 177 | C G T A G C T A C G A T C T A G A C G T G T C A C G T A C G T A A G T C C G T A T G C A T A C G | FoxL2(Forkhead)/Ovary-FoxL2-ChIP-Seq(GSE60858)/Homer | 1e-2 | -6.173e+00 | 0.0052 | 1458.0 | 15.09% | 5478.6 | 14.06% | motif file (matrix) | svg |
| 178 | G A C T C T A G C T A G A G T C T G C A A C T G A C G T A C G T C T A G T C A G | AMYB(HTH)/Testes-AMYB-ChIP-Seq(GSE44588)/Homer | 1e-2 | -6.136e+00 | 0.0054 | 2265.0 | 23.44% | 8657.3 | 22.22% | motif file (matrix) | svg |
| 179 | A G T C G A C T C A G T G T A C A G T C A T C G T C A G A C T G G T C A C G T A | Stat3(Stat)/mES-Stat3-ChIP-Seq(GSE11431)/Homer | 1e-2 | -6.047e+00 | 0.0058 | 711.0 | 7.36% | 2582.7 | 6.63% | motif file (matrix) | svg |
| 180 | C T A G C A T G C A T G A G C T G A C T C G T A G A T C A G C T G T C A A T G C T C G A C T A G C A T G A C G T A G T C C T G A | LXRE(NR),DR4/RAW-LXRb.biotin-ChIP-Seq(GSE21512)/Homer | 1e-2 | -6.018e+00 | 0.0060 | 68.0 | 0.70% | 190.8 | 0.49% | motif file (matrix) | svg |
| 181 | G A C T A G C T G T A C G A C T C T G A A C T G G T C A C T G A A T G C T A C G G A C T A C G T A G T C G A C T C T G A | HRE(HSF)/Striatum-HSF1-ChIP-Seq(GSE38000)/Homer | 1e-2 | -5.993e+00 | 0.0061 | 341.0 | 3.53% | 1178.3 | 3.02% | motif file (matrix) | svg |
| 182 | G C A T G C A T C T G A A C G T C T G A A C G T C G T A C G T A C G T A A G T C G T C A G T C A | Foxf1(Forkhead)/Lung-Foxf1-ChIP-Seq(GSE77951)/Homer | 1e-2 | -5.870e+00 | 0.0068 | 1578.0 | 16.33% | 5962.4 | 15.30% | motif file (matrix) | svg |
| 183 | C G A T G A C T C G A T T C A G G A C T A C G T C A G T C T G A G A C T G A C T A G C T C G A T A C T G A T C G G T A C G C T A | NF1:FOXA1(CTF,Forkhead)/LNCAP-FOXA1-ChIP-Seq(GSE27824)/Homer | 1e-2 | -5.859e+00 | 0.0069 | 87.0 | 0.90% | 256.2 | 0.66% | motif file (matrix) | svg |
| 184 | T A C G C T G A T C G A C G A T C T A G C T A G T C G A C T G A T C G A T C G A C G T A T C G A G C A T C A T G C G T A T A C G G C A T T G A C C G T A A G C T | NFAT:AP1(RHD,bZIP)/Jurkat-NFATC1-ChIP-Seq(Jolma\_et\_al.)/Homer | 1e-2 | -5.473e+00 | 0.0100 | 259.0 | 2.68% | 883.4 | 2.27% | motif file (matrix) | svg |
| 185 | C T A G A C G T A G T C C G T A A C T G A G T C G C A T A C T G G C A T A G T C G A C T G A T C G C A T A G T C A G C T | ZNF317(Zf)/HEK293-ZNF317.GFP-ChIP-Seq(GSE58341)/Homer | 1e-2 | -5.411e+00 | 0.0106 | 148.0 | 1.53% | 477.7 | 1.23% | motif file (matrix) | svg |
| 186 | C G T A C T G A C T A G C G T A C G T A A G T C C G T A C A G T G C A T G T C A C G A T A C T G A C G T G C A T G A T C | PGR(NR)/EndoStromal-PGR-ChIP-Seq(GSE69539)/Homer | 1e-2 | -5.408e+00 | 0.0106 | 366.0 | 3.79% | 1286.0 | 3.30% | motif file (matrix) | svg |
| 187 | T C A G T A C G G A T C G T A C T C G A C G A T C T G A C G T A G C T A C G T A | Hoxd11(Homeobox)/ChickenMSG-Hoxd11.Flag-ChIP-Seq(GSE86088)/Homer | 1e-2 | -5.296e+00 | 0.0118 | 3802.0 | 39.35% | 14830.7 | 38.07% | motif file (matrix) | svg |
| 188 | G T A C G A T C C A G T A G T C A G T C A G T C T G C A G A T C C T G A A T G C G T C A A C G T | WT1(Zf)/Kidney-WT1-ChIP-Seq(GSE90016)/Homer | 1e-2 | -5.229e+00 | 0.0125 | 694.0 | 7.18% | 2543.1 | 6.53% | motif file (matrix) | svg |
| 189 | T A G C G T A C C G T A C T A G A C T G T G C A C G T A A T G C C G T A A T C G | AR-halfsite(NR)/LNCaP-AR-ChIP-Seq(GSE27824)/Homer | 1e-2 | -5.214e+00 | 0.0127 | 5496.0 | 56.88% | 21655.6 | 55.59% | motif file (matrix) | svg |
| 190 | C G T A C G A T G A C T G A C T T G A C C T G A A T G C C T G A T A G C A G T C A C G T T C G A C A T G T A C G G A C T A T C G G A C T A C G T C T G A T C G A C G T A | Brachyury(T-box)/Mesoendoderm-Brachyury-ChIP-exo(GSE54963)/Homer | 1e-2 | -5.181e+00 | 0.0130 | 415.0 | 4.29% | 1476.5 | 3.79% | motif file (matrix) | svg |
| 191 | T A C G G A C T T G A C C G T A A C G T G A T C G T C A C G T A A C G T A T G C C G T A G A C T | HOXA2(Homeobox)/mES-Hoxa2-ChIP-Seq(Donaldson\_et\_al.)/Homer | 1e-2 | -5.175e+00 | 0.0130 | 174.0 | 1.80% | 575.2 | 1.48% | motif file (matrix) | svg |
| 192 | C T A G C T A G C G T A C G T A T A C G C G A T C T A G C T G A C T G A C G T A T A C G G A C T | PU.1:IRF8(ETS:IRF)/pDC-Irf8-ChIP-Seq(GSE66899)/Homer | 1e-2 | -5.102e+00 | 0.0139 | 303.0 | 3.14% | 1055.0 | 2.71% | motif file (matrix) | svg |
| 193 | T G A C C T A G T C A G G T C A C G T A T C A G C G A T T C A G T C G A T G C A C T G A T A G C | PU.1-IRF(ETS:IRF)/Bcell-PU.1-ChIP-Seq(GSE21512)/Homer | 1e-2 | -4.811e+00 | 0.0186 | 2091.0 | 21.64% | 8041.2 | 20.64% | motif file (matrix) | svg |
| 194 | C T A G T A C G G A T C G T C A T C G A A C G T T C A G C G T A C G T A C G T A | Hoxd10(Homeobox)/ChickenMSG-Hoxd10.Flag-ChIP-Seq(GSE86088)/Homer | 1e-2 | -4.639e+00 | 0.0219 | 1847.0 | 19.11% | 7085.3 | 18.19% | motif file (matrix) | svg |
